# Observations on the Epidemic Yellow Fever of Natchez, and of the South-west

**Published:** 1841-12

**Authors:** John W. Monette

**Affiliations:** Washington, Mississippi


### WESTERN JOURNAL

OF

### MEDICINE AND SURGERY.

---

DECEMBER, 1841.

---

Art. I.—*Observations on the epidemic Yellow Fever of Natchez, and of the South-west.* By John W. Monette, M. D. &c., of Washington, Mississippi.

(*Continued.*)

### GENERATION OF YELLOW FEVER MALARIA.

The advocates for the local origin of Yellow Fever in the United States, are not unanimous as to the exact circumstances under which the malaria, or infectious air of Yellow Fever is generated. The only point on which they appear to agree, as a general principle, is, that it is the result of the solar heat upon some putrescent matter. This may be any variety of animal or vegetable matter, in a state of decomposition; or it may proceed from both those classes of matter combined; or it may be the combined gases from each of these separately, which produces the malaria necessary to induce epidemic Yellow Fever. Rarely do we find any two of the advocates for the local domestic origin, who agree as to the *particular kind* of animal or vegetable matter, which is most productive of the miasm necessary for producing an epidemic. Some ascribe it chiefly to one kind; others to a different kind. Some require the aid of vegetable decomposition, with *marsh-miasm;* others can, satisfactorily to themselves, show that it is the product of *animal* matter chiefly.

The advocates of this doctrine range themselves under one of each of the following sources of Yellow Fever Miasm:

1. Animal putrefaction.
2. Vegetable putrefaction.
3. Marsh-miasmata.
4. Sensible changes in the atmosphere.

Others ascribe the disease, when epidemic, to some peculiar atmospheric constitution, or some mysterious combination in the elements, entirely beyond our comprehension, the *to thion of Hippocrates.*

We propose to note each of these briefly, and to pass on to what we consider a more rational and consistent explanation of the origin and production of Yellow Fever malaria. We design to show that none of these local causes are adequate to the production and dissemination of this epidemic malaria; and that we must extend our researches beyond such matters if we wish to arrive at truth, and to protect our ports from this pestilential disease.

1. Animal Putrefaction.—Those who look to this source, conceive that this poisonous miasm is thrown off into the atmosphere from either of the following animal matters, viz: From putrid or putrescent fish, putrid bacon or pork, putrid animal carcasses near cities,* from putrid oysters in cellars, or in piles in the open air, from putrid hides in ships and on the wharves, from the opening of old burying grounds, &c.

That none of these are *essential* sources of Yellow Fever mimiasm, has been established almost beyond controversy.

*Dr. Cartwright, of Natchez: See Medical Recorder, vol. ix, p. 5 to 10 and p. 228.

Dr. Bancroft, in his great work on marsh-miasm and Yellow Fever, has adduced an array of facts and evidence incontrovertible, proving that animal putrefaction may exist to any extent, and under *all circumstances*, without in any wise exciting Yellow Fever; and, consequently, that it is not a *necessary* source of Yellow Fever miasm; and which, of course, must proceed from some *other cause.* He has shown, conclusively, that this species of putrefaction is *not a frequent* concurring circumstance, where Yellow Fever has originated He has also shown that animal putrefaction, to a great extent, has existed in cities and other localities, under the most favorable circumstances, for the production of this miasm, according to the putrefactive theory, and still no Yellow Fever has been produced either sporadically or epidemically. He has further shown that putrid pork, putrid fish, putrid bacon, &c., will not generate any pestilent miasm. Nor will dead bodies of any kind, in a putrescent state, produce Yellow Fever, or any other malignant disease, not even human bodies putrefying by hundreds on the burning sands of Egypt. Neither is it produced from the intolerable stench exhaled from factories of glue and catgut; nor from tanneries, slaughter-houses, tallow-chandleries, &c. Indeed those engaged in these avocations are generally blest with the most perfect health; and their business appears to confer a partial immunity from ordinary disease.

These facts are incontrovertible. They establish the doctrine sanctioned by experience, that the miasm or morbific malaria, or the infection of epidemic Yellow Fever, whatever be its nature and constituents, is not in any sense connected with, or dependent upon the *fœtor* of animal putrefaction in any of its stages of decomposition.

Again it has been placed beyond doubt, by Dr. Bancroft and others, that Yellow Fever infection has been generated, or accumulated abundantly in ships at sea, where no putrid matter, either animal or vegetable have existed as a *possible* source. A case of this kind in point is found in the United States brig "Enterprise," more particularly noted hereafter. On the other hand there are towns and ports, within the tropics, where putrid vegetable and animal matters abound, in combination, with the offensive effluvia from filthy and muddy bottoms of shallow harbors, and yet the people of these places enjoy almost uninterrupted health. The town of Campeachy is an example hereafter cited.

We shall show also in some of the most destructive epidemics of Natchez and other south-western towns, that animal putrefaction had no agency whatever in its production. In fact, that putrefaction, in any form, is not an essential agent in producing epidemic Yellow Fever.

### 2. VEGETABLE PUTREFACTION, OR DECOMPOSITION.

Those who ascribe it to vegetable effluvia, trace it to putrid coffee, putrid potatoes,* putrid oranges, rotten corn, putrid sour-krout,† sour porter in warehouses or near the wharves.

That this is not an essential cause of yellow fever miasm, is proven by the same array of facts and arguments presented in the work of Dr. Bancroft, against animal putrefaction.

Vegetable putrefaction, of the most offensive kind and in the most extensive degree, has occurred without producing a single case of yellow fever in those exposed to the effluvia. On the other hand, the most desolating epidemics have prevailed where no mass of vegetable putrefaction could be found.

In every county and settlement throughout Mississippi and Louisiana, how often do we find hundreds of bushels of cotton-seed in a rotting state, exhaling its intolerable stench, while no disease of any kind, much less yellow fever, is produced in those daily exposed to its influenc? These immense accumulations of decaying cotton seed are found on almost every plantation, exhaling a most offensive fœtor, to which every soul on the plantation is more or less exposed during the burning suns of summer as well as after the rains of

---

* Dr. Rush: See Medical Inquiries.

†Dr. Cartwright: See Medical Record, Vol. 9, p. 226.

spring and autumn. Yet we never have known, or heard of any case of disease, much less yellow fever, traced to such source.

For many weeks before the epidemics of 1837 and 1839, in Natchez, the most zealous advocate of vegetable origin, did not even suspect any matters of that kind; for nothing of the kind existed about the city, as *all admitted.*

On the other hand, after the great tornado of May 7th, 1840, the whole city of Natchez was filled with the ruins of demolished buildings, and every kind of matters covered up in the masses of ruins within the city: the forest growth in and about the city was a scene of destruction; and immediately opposite Natchez, a large grove of cotton-wood and other trees, covering probably near sixty acres, was entirely torn to atoms, covering the whole surface to the depth of many feet, with the mass of timber, trunks, branches and foliage, of a most luxuriant growth. This was in the very midst of the process of vegetable decomposition in July and August following, after the subsidence of the river inundation.

Besides this, at the base of the bluff near the Natchez landing, there was a pond covering about ten acres, with a common depth of 3 or 4 feet water. This pond was filled to the bottom, and for a foot above its surface, with ten thousand fragments of demolished houses, with carcasses of animals, and even men who were supposed to have been buried under them. In this condition it remained, frequented by hundreds of people every day, until October, exposed to all the effluvia which could be exhaled by a powerful sun for nearly four months. With all these extraordinary circumstances, Natchez enjoyed uninterrupted health through the whole season; contrary to the confident prediction of a most desolating yellow fever epidemic, by the advocates for the origin from such causes. New Orleans was free from yellow fever that autumn.

In ordinary seasons, when vegetable decomposition, as a matter of course, would progress more rapidly, with alternate showers and sunshine, we never have any apprehension of yellow fever in Natchez: but when all moisture is dissipated from the surface of the earth, and when vegetable decomposition necessarily ceases for *want of moisture,* then yellow fever begins to make its appearance, if at all. Yet the yellow fever of Natchez has been ascribed at different times to diverse vegetable matters, because once or twice such matters were found soon after yellow fever made its appearance as an epidemic.

### 3. MARSH MIASMATA.

Those who ascribe yellow fever to marsh-miasmata, have traced it in imagination, to the exhalations from stagnant water in marshes; to the effluvia from marshy grounds, when all moisture is nearly dissipated, and the dried surface begins to crack open; to the exhalations from mud and filth in the shoal harbors, or on the slimy banks, and battures of great rivers; to common city filth in sewers, back yards and alleys; to loose earth recently thrown up from beneath the surface;* and when these are wanting, to whatever their fancy may suggest. All these we hope to show are *non-essential* in the yellow fever epidemics of Natchez.

Dr. Bancroft, in his great work on yellow fever, has labored hard to prove that *marsh-miasm* is the true and essential cause of yellow fever. On this point his labor has all been in vain. The experience of thirty years since repudiates his doctrine.

We find from medical records too numerous to cite, that this terrible disease in the West Indies, prevails equally in the vicinity of marshes, and on the most baren rocks, where no marsh is near. We find it equally prevailing on high and low situations, upon the Natchez bluff, 300 feet above tide, and on the low alluvions of Charleston and New Orleans, scarcely above high tides; on the sandy beaches of Mobile and Pensacola, and upon the barren rocks of Curacoa in the West Indies,

*Dr. Menvill: North American Journal of Medical Science, vol 1, p. 1 to 20.

and of Lisbon, Cadiz, and Gibralter, in Europe. We find it likewise prevalent on the deltas of the Mississippi, Oronoco, and the Amazon.

On the other hand, we find in many of the low marshy regions of Louisiana, in Attakapas, Terre Bonne, La Fourche Interior, and about St. Augustine, in Florida, the most healthy population of all the South, where longevity is proverbial,* and Yellow Fever is unknown.

In the West Indies it is endemic all the year in the *commercial ports* on the coasts, while it is unknown in the *interior* towns of the islands. It is equally unknown in the Southern towns of the United States, where there is no direct trade with West India, or infested ports.

The most extensive marshes in the Southern portion of the United States, are as free from Yellow Fever as are the pine hills of Mississippi and Alabama. This is confirmed by all experience in the extensive plantations which everywhere spread over the Mississippi bottoms.

### 4. SENSIBLE CHANGES IN THE ATMOSPHERE.

Those who ascribe epidemic Yellow Fever to this cause, have conceived that the disease was produced by a dry and heated state of the atmosphere; or by moisture in the air at a high temperature. Professor Caldwell contends that the predisposition to Yellow Fever is induced by a continued mean temperature of 80° Fahrenheit, for forty days successively.† Others believe that sudden vicissitudes, from hot to cold, and the reverse, produce the epidemic constitution of the air. Others ascribe it to the electric condition of the air as indicated by the amount of thunder and lightning which is observed during such periods.

That this disease does not originate essentially from a *humid* or *arid* state of the atmosphere is clearly shown by the

*See Dr. Cartwright on Jussiuea Grandif., West. Jour. of Med. and Surgery.

†See Med. and Phys. Memoirs, Lexington, 1826, p. p. 141, 142, &c.

whole history of Yellow Fever epidemics. This disease has been epidemic in cities of the United States and Europe as well as in the West Indies. It has made its first appearance in Philadelphia, in the hottest and driest weather, and it has begun to spread in New Orleans, *after* a dry summer, while the earth was washed with showers almost every day. It has likewise made its first appearance in Natchez in the most hot and sultry condition of the air, after the greatest drought throughout the whole State, and it has spread and continued to prevail through all the vicissitudes of weather above a temperature of 40° of Fahrenheit's thermometer. It has also begun to spread in Natchez and in New Orleans under all these vicissitudes of weather. It has prevailed in these two cities after every variety of weather during the spring months; after a wet and dry spring; after a late cold spring, and after an early and warm spring.

It prevails at times in *commercial* ports where the air is either habitually dry or humid; no less on the dry and parched shores of Curacoa, than on the flat alluvial marshes of New Orleans. But in all places where it has ever prevailed as an epidemic, it has been after a long dry oppressive summer; and this is especially true in relation to its epidemic visitations in the United States.

Dr. Peixotto informs us that the Yellow Fever is endemic in the island of Curacoa during the year, at all times and seasons; but that it prevails more especially in the "*calm and sultry months;*" that "*new comers* from Northern latitudes are its *appropriate subjects;* the *natives* and *seasoned inhabitants being exempt from its attacks.*"* He moreover informs us that "it never assumes an epidemic character unless after the arrival of large numbers of *northern strangers* during the *sultry* months of the year."

On this island, situated under a vertical sun, the climate is hot, but the air of the island is proverbial for its *dry elastic* qualities. He informs us, "the air is *pure* and *dry*, and sel-

*See New York Med. and Phys. Jour., vol. 1, p. p. 411, 412, &c.

dom, or never, darkened with mists and fogs, so frequent at the north;" that "during the droughts which are common, the leafless desolation of winter reigns under a tropical sun; ordinary wells are exhausted; animate and inanimate nature suffers under the burden, and seems nearly ready to waste away and perish."* On this island there is no marsh and scarcely a living stream of fresh water.

This is one of the islands where Yellow Fever is *indigenous;* it is also *indigenous* to *certain ports* in numerous other islands in the Caribean sea, and in the Bahama and other groups, together constituting the West Indies, and the Great Antilles. Here is the proper orgin of the American Yellow Fever.

The views of Professor Caldwell, before alluded to, as to an unremitted mean temperature at or near 80° of Fahrenheit, approach nearer the true cause, than any thing we have seen. This degree of mean temperature, provided the atmosphere be calm and sultry, is requisite, to a certain extent, to prepare the atmosphere of a port or city for the prolific spread of *yellow fever infection,* when once introducd. But in the United States, this degree of mean temperature has been witnessed, where no yellow fever has been produced or existed in any condition; whereas we know that yellow fever has prevailed as an epidemic, *un*preceded by a mean temperature of 80° for 40 days. Our own city of Natchez will furnish the facts without going further.

According to the meteorological table given by Dr. Parlee in his account of the yellow fever of 1817 and 1819, we find as follows,† viz.: That the *mean* temperature of July and August for sixty-two days, in the years 1814, 1816, and 1818, was steadily from 80° to 83°, and yet no yellow fever occurred in Natchez in either of those years. In the year 1817, the mean temperature during July, for 31 days, was 80°—and during August, for 31 days, it was 77°—during the month of September, for 30 days, the mean temperature was

*See New York Med. and Phys. Jour., vol. 1, p. p. 398, 400.
†See Chapman's Med. and Phys. Jour., vol. 3, p. 17, &c.

only 73°—and yet the yellow fever prevailed with great mortality, having become epidemic not until the 28th of September. Again, in the following year of 1818, the mean temperature during the months of July and August, was for each, 82°—being 60 days steadily at 82°—and yet no yellow fever appeared in Natchez that year. Again in the following year of 1819, the mean temperature of July and August was for each, 79°—being for 62 days. For September following, the mean temperature was only 75°—and yet the yellow fever prevailed with great mortality; having become epidemic about the middle of September.

We might cite authority to any extent to show that the same state of atmosphere has existed under similar circumstances elsewhere. But we will make only one more reference to the tables of Dr. Tooley of Natchez, and he is proverbial for his accurate observations. In the year 1824, the mean temperature for July was 86° for 31 days; and for August 82½° for 31 days; thus giving an average mean heat of 84°—yet no yellow fever was known in the city. In the year 1825 the mean temperature for the month of July was 81° for 31 days; and for August 83½° for 31 days. Thus the general mean temperature was up to 82° for 62 days. This year the yellow fever prevailed with great violence as early as the 20th of August, or after 52 days of that mean temperature.

Thus it would appear, that although a continued high temperature is a necessary circumstance in causing an epidemic yellow fever, that *something else* super-added is requisite. In the year 1824, there was a less number of rainy days in August than in August of 1825*—so that a deficiency of rain is not the cause exclusively.

If it were owing to a deficiency of moisture in the air, this could easily be obviated by watering the streets, repeatedly, as often as evaporation was complete. *Heat* is necessary to the generation and dissemination of yellow fever infectious

*In 1824 there was rain 12 days in July; 9 in August. and 5 in Sept.
" 1825 " " " 12 " in July; 11 in August, and 5 in Sept.

air; but heat, if attended with much wind and agitation of the atmosphere, will not generate and diffuse this infectious air through a city.

At certain times there certainly is more or less of a general "epidemic constitution of the atmosphere" over an extensive scope of country. This peculiar condition does, doubtless, predispose the population in crowded cities and ports to certain grades of fever, and to yellow fever especially. But as to yellow-fever "epidemic constitution," we believe there is nothing very peculiar; nothing that can properly be called the *to thion* of Hippocrates, or the "*seminarium e cœlo dimissum*" of Diemerbroeck. We believe that it is simply a condition of the air, which we can reasonably conceive of; and concerning which we may safely predicate a course of conduct, which will prevent, or protect a city from, an epidemic yellow fever.

This is simply a hot, sultry condition of the general atmosphere, whereby the circumscribed air of a town or city becomes charged with human and other effluvia, which become more cencentrated by the absence of proper ventilation and change of air, until it becomes a fit *nidus*, or receptacle, for the reception and dissemination of what is properly *Yellow Fever infection.*

### YELLOW FEVER IS A COMMERCIAL DISEASE.

This is a point already established, and it is necessary here only to illustrate that point from authority and by exemplification.

Yellow Fever, as an epidemic, is a disease peculiarly confined to seaport towns and cities in the United States; or to those places contiguous to which it has been transported in one form or another. It is found prevalent chiefly in seaport towns and cities in the Southern portion of the United States. In those maritime towns, which carry on *no trade* with the West Indies, or with infected cities near the Atlantic seaboard, we find the people are exempt from this malignant disease. The interior towns which, by their location and natural circumstances, are debarred from direct water communication with the large commercial ports, and, from the West India trade, are uniformly exempt from all appearance of this disease.

So true is this position, in the United States, that we scarcely deem it necessary to confirm it by the citation of authority. The whole history and records of the medical profession, relative to the appearance of this disease as an epidemic, show conclusively that its ravages have been primarialy in commercial ports, where there are frequent arrivals of vessels from West India ports and seas. Where it has appeared in towns which were not ports it can be shown that the disease was transported thither by persons already infected or by other means.

As Dr. Ramsay,* of Charleston, remarks, "Yellow Fever is eminently the disease of cities." It proceeds from an atmospheric contamination, which is perfectly *harmless* to natives of the West Indies, and to those who have become seasoned or acclimated to it; but is poison and death to strangers with northern habits and constitutions.

Dr. Rush, with all his zeal to establish the local origin of Yellow Fever miasm, from putrescent vegetable matters, and from city filth, could not resist the evidence, forced upon him, that ships sometimes contained in their holds, a poisonous air which could and often did produce yellow fever about the wharves. Pressed on every side by such indubitable evidence, it is surprising to what additional sources of local effluvia he resorted, rather than abandon his favorite theory of local origin. After the epidemic of 1793, which he ascribed to putrid coffee, every visitation presented new apparent sources of miasm, as if to show the fallacy of his theory. Such were the diversity of circumstances, under which subsequent epidemics appeared, that he was compelled, in order to sustain himself, to *make numerous additions* to the former list of causes. To adapt his theory to the changing *phases* of circum-

*See Med. Repos., vol. 4, p. 100.

stances, he is compelled to admit the *combined influence* of docks, sewers, filthy gutters, filthy yards and cellars; also every variety of animal and vegetable putrefaction, and, lastly, partially to admit, to a certain extent, the *real source*, which he denominated "*the foul air of ships*," diffusing itself into an atmosphere vitiated with exhalations extricated by "*great solar heat.*" Not only this, but he was obliged to deny the peculiar character of the disease, and gave it the name of "Bilious Yellow Fever."* With such arguments did he lead the judgments and prejudices of the people of Philadelphia, and of many theoretical physicans.†

Numerous instances were presented in the little trading towns and ports on the Delaware river and at different points on the Chesapeake where yellow fever had been introduced, without the sources to which Dr. Rush ascribed it in Philadelphia, and in which he was obliged to admit that "*the foul air from the ships acted as an* EXCITING CAUSE." This ground was taken by those of the same school; and they admitted and contended that this "*exciting cause*" was frequently introduced in the vessels: and recommended such vessels to be quarantined, &c. Yet they still adhered to their former belief that this was not *contagion*, which appears only a specious term to cover their defeat. Dr. Rush finally was compelled to admit that a virulent infection could be, and had been, introduced by ships in the form of "*fomites*" in clothes, bedding, and in trunks, boxes, &c.

The same doctrine was advocated by "The Academy of Medicine, in Philadelphia." In a letter to Gov. Mifflin, of Pennsylvania, Dr. Philip Syng Physick, the President of the Academy, by order, unequivocally admits, and contends, that the yellow fever of 1798, in Philadelphia, was in part to be ascribed to "*the foul air discharged* from the ships Deborah and Mary," from St. Domingo—which island they left respectively on the 18th and 29th of July. Again he declares: "We

*See his "Observations on Yellow Fever," addressed to the citizens of Philadelphia, in 1799, A. D. Also Med. Repos., vol. 3, p. 294-5.
†Also, Med. Repos., vol. 6, p. p. 156-7 and 162.

are the more determined in our opinion of the foul air of the Deborah and Mary, being the cause of many cases of our fever, from *similar cases of fever having been often produced* from similar causes; *instances of which* we mentioned in our letter to you last year."* Again he says: "To guard against *the frequent source of yellow fever*, from the *noxious air in the holds of the vessels*, we recommend the unlading of such vessels, as contain cargoes liable to putrefaction, and the discharging of the *ballast of all vessels* at a distance from the city, during the months of June, July, August, September, and October."†

In this and numerous other instances which might be cited the fact that vessels do, and have repeatedly brought yellow fever into ports and trading towns along the seaboard, is clearly admitted by Dr. Rush and other distinguished advocates of *local origin;* the fact of *importation* is established by their own testimony. Yet they rest their whole argument in the controversy upon the unimportant question: whether this pestilential air is solely the result of *human contagion*, or *personal infection*, in contradistinction, to an infectious air produced by certain natural causes?

To be clearly understood as we progress in our remarks, we conceive the following to be a good definition of *contagion* and *infection*, viz:

Contagion is a poisonous material, capable of exciting a peculiar disease in healthy bodies exposed to its influence; and emanating with *that capacity* or power, at all times and under all ordinary circumstances, from a body labouring under that peculiar disease.

Infection is some noxious gaseous matter, capable of exciting certain kinds of fever, *and not emanating in that form;* having the power of exciting the disease, from some properties assumed after it had emanated from a diseased body. Such is the infection of yellow fever.

Whether yellow fever can be introduced and disseminated

*See Medical Repository, vol. 2, A. D. 1800, p. 327 to 331.

†Ibidem.

in a healthy port by vessels from infected West India ports, is a point which many yet deny. The evidence, however, in favor of importation is clear and conclusive, to those whose judgments are open to impartial testimony. There is a certain condition of atmosphere in cities and ports at times in the United States, when yellow fever infection will *not* readily spread and produce an epidemic. This is when the air is too pure, or too cool to disseminate the infection from a single point. There are other conditions of air in towns and cities, during the hot sultry season of summer and autumn, when this infection has spread rapidly from one or more points, and speedily induced an epidemic. These facts are well established. Even Dr. Rush, and many of his cotemporary disciples in the doctrine of domestic origin, admit that vessels have become thoroughly infected, and have communicated yellow fever to those about the wharves where they laid. True they contend that this infection was produced by some putrescent matters in the vessel or upon the wharf. But as to the fact of *the vessel being infected*, they entirely agree. If one vessel become infected, a dozen may. If one vessel can spread a malignant disease in a certain number of the population, a greater number of infected vessels may certainly spread that disease *more extensively.*

In adducing authority on this point we claim the full weight of their testimony with all impartial inquirers—because we have been careful to exclude the testimony of those who rank with contagionists. We have been careful to select the facts and admissions of those who are advocates of the theory for the local and domestic origin of yellow fever epidemics.

All testimony tends conclusively to establish the point, that ships, which have been long at sea in tropical latitudes, have occasionally become thoroughly infected with the miasm or infection proper of yellow fever, either while at sea or while in some West India port; that this infection has become so concentrated as to produce yellow fever in its most aggravated forms, in those who have gone aboard from our shores; that such persons have taken genuine yellow fever and died with it, in five or ten days, after they first breathed the infected air of the ship, although at that time none of the sailors or hands on the ship, were laboring under that disease. In this manner doubtless, in some instances, has yellow fever begun to prevail in sea-ports, among the many who daily, unconscious of the infection, visit such ships while in port. In this manner, in many instances, have the first twenty or thirty cases been traced to intercourse with an infected ship, or to goods from the same. These first cases are generally persons near the wharves, draymen, day-laborers, and others who are engaged in assisting to unload the cargo. It is perfectly immaterial whether the ship brought the infection from a *foreign port,* or generated it during her voyage. If the air in her hold can be disseminated about the wharf, or even if it will cause those who visit the ship to die of yellow fever, it certainly is an *imported* disease to those who suffer.

A ship can certainly become infected, and much more completely than a house, or a district of a city. On shipboard, the air below is necessarily close for want of ventilation. We will not travel over all the mass of testimony which might be adduced: but will cite only a few instances in our own country, which are conclusive: for one unquestionable case, is as amply illustrative of the principle, as twenty.

Dr. Tully,* one of the most scientific physicians of Connecticut, gives an account of a number of cases of yellow fever at Knowles' Landing, in August, 1796. This is a village on the Connecticut river, about six miles below Middletown, containing a population of about two hundred souls, and situated on a steep declivity, with spacious and airy streets, and not crowded with houses. The number of cases that occurred at this place was eleven, of whom nine died. Every case was clearly traced to communication with a vessel which had recently arrived from Havana, on board which one of the sailors had died with yellow fever on the voyage. The whole number of cases occurred and terminated in the

*See New York Med. and Phys. Jour. vol. 1, p. 153–8.

course of a fortnight; for the alarm excited by the appearance of a malignant disease among them, caused a complete and speedy desertion of the village, and non-intercourse with the ship. This village has always maintained the character of uncommon salubrity. Up to the arrival of the infected ship, no disease of any kind prevailed; and immediately on the ship being abandoned by the inhabitants, the disease ceased. The infection on board this ship was not generated by putrefaction of either animal or vegetable matter, as none existed on board; and Dr. Tully declares that she was perfectly clean; no such matters were found in the town, or suspected by any as the cause of the disease: the ship was the undoubted source, and none were attacked but such as had been on board. In this case had the weather been such as generally *precedes epidemic* yellow fever; and had the inhabitants not fled, but remained, and continued their intercourse with the infected ship, the town would doubtless have been visited with *epidemic yellow fever*.

Dr. Tully informs, us that many other cases occurred at the different places at which this vessel anchored in ascending the river, and always in those only who had been on board. He also informs us, that, for the last twenty-five years, scarcely a year has passed, in which one or more similar cases have not appeared at different points on the Connecticut river; all of which are clearly attributable solely to intercourse with vessels from the West Indies, or from Southern ports of the United States.

Dr. Bayley,* health officer of the port of New York, gives an account of a number of cases of yellow fever which occurred in the autumn of 1821, at the "quarantine establishment," on Staten Island, six miles below the city. From the 8th of September until the 7th of October, twenty-nine cases and twenty-one deaths occurred; of the latter fourteen had black vomit. These were clearly traced to intercourse with infected vessels then lying at the wharf, and recently from the West Indies and New Orleans. The health of those about

*New York Med. and Phys. Jour., vol. 1, p. 27, 28, &c.

the "quarantine establishment" never was better, both immediately before and after these cases: nothing like bilious or remittent fever had been seen: neither marshes, nor filth, nor vegetable putrefaction existed any where in the vicinity. The cases were traced clerarly to the ships, and to them alone; and each case occurred just five days, and one on the sixth day, after the particular exposure to the infected air of the vessels. A washerwoman and her two daughters took the disease, without having been on board the vessels. They contracted their disease by handling and washing the foul clothes and bedding of four men who had died of yellow fever, about four days previously. The bedding and clothes had been thrown aside until taken to her to be washed. She took the disease just five days after she had handled the clothes, and died on the fifth day of her disease; her daughters were attacked afterwards. In this case a favorable condition of the air would, no doubt, have caused it to become epidemic.

Dr. Bayley* also gives us the case of the United States' brig Enterprise, infected with yellow fever, at the "quarantine ground," in 1822. This vessel was perfectly clean, and free from any animal or vegetable putrefaction. She arrived from Havana with ten cases on board; and immediately the sick were removed to the hospital, and the well were quartered on shore to avoid the infected air of the vessel. She was then thoroughly cleansed, ventilated, washed, and whitewashed with lime, in a tenfold degree; lime was slaked in her timbers in large quantities. Yet, after this purification, she retained the infection, and communicated the yellow fever to those who afterwards went on board; of whom five, out of eleven, died. The process of purification was again instituted. Artificial ventilation with windsails was constantly performed; water to the depth of several feet, was daily let in and pumped out: lime was strewed in the hold, and her timbers thoroughly whitewashed, and still the infection was not destroyed until cold weather. In this case, her own crew having taken

*New York Med. and Phys. Jour., vol. 1., p. 426–7, &c.

the disease first, the people from shore avoided the vessel and escaped the disease.

Another case is given by Dr. Kollock, and quoted by Dr. Rush, in which a vessel at sea, in tropical latitudes, engendered on board a malaria which finally produced yellow fever. This is the case of the United States' frigate General Greene, which became infected while on a cruise in the West India seas, and did not become disinfected until she reached the cold climate of Rhode Island. During the time she retained the infection, every kind of cleansing, fumigation, and ventilation, were used freely, but ineffectually. In this case, the vessel was new, and perfectly clean and healthy, when she left Newport, Rhode Island, on the 3d of June, 1799. She had on board a complement of two hundred and fourteen souls besides large quantities of provisions for a cruise in the West India seas. Having encountered a storm, soon after she put to sea, the vessel became leaky, and a noxious malaria was generated, during a subsequent period of unusually *hot weather*. At first, and for some days, the disease assumed the symptoms of a violent bilious fever, with no deaths until they arrived in the port of Havana; when immediately several cases began to assume symptoms of *yellow fever*. "After this period," says Dr. Kollock, "three, four, and five new cases occurred daily; and the violence of the symptoms seemed to *increase with the multiplication of cases*, during the six days she lay in port."* Cases and deaths continued to multiply daily, until she passed the capes of Virginia, when the disease became gradually milder. The whole number of the cases was forty; and twenty of them died. In this case, an infectious air was generated in the ship's hold, by the number on board, during the hot weather and leaky condition of the vessel; and it appears that the "leaven" of infection was superadded at the port of Havana, either by persons going on shore, and contracting the disease there, or by the introduction of infected air, &c. Many other cases might be cited, in

*Med. Rep., vol. 4, p. 3.

which infection has been generated and carried in ships into ports, and there produced yellow fever in those from shore who entered on board, although the crew of the vessel, being acclimated, remained free from disease.

We will here make a few remarks again upon the generation of miasm on board ships in tropocal climates. The confined air in the holds of ships is more likely to be exhausted by respiration, and charged with human effluvia than even a city; especially where there are many souls on board, who are often confined below on occount of storms and winds. In them thorough ventilation is extremely difficult, and the temperature is almost constantly up to the miasm point. The infectious air is thus rapidly formed in many cases, and in this state, a vessel arriving at a port where yellow fever prevails, will be ready to receive the leaven from the infected air of the city, either by the crew visiting the infected districts on shore or by the introduction of the infection in the form of *fomes* in goods. The United States frigate General Greene, before referred to is a good illustration.

The temperature at sea in tropical latitudes is seldom below 80° of Fahrenheit, and often from 90° to 100° according to the degree of reflection and concentration of the sun's rays. By a register kept by Dr. Thos. Rodman,* embracing thirty observations between the first of November and the fifth of December, at 12 o'clock, M., each day, between latitude 22° north, and 22° south, it was ascertained that the lowest temperature of the air was 79° and the highest in the shade 86°. The lowest temperature of the sea below the surface was 78°, and the highest 84°. The mean temperature of the air during that time, at 12 M. was 83°, and of the water 81.° Thus the temperature within vessels while in the tropics cannot be low enough to *destroy* or neutralize miasm, much less infection: the former requiring a temperature of 50° and the latter of 32° Fahrenheit. Thus however slowly it may be generated, it continues to accumulate until the ship enters a northern climate. This register was kept during a cool healthy sea-

*Vide Coxe's Medical Museum, vol. 1, p. 83–4.

son, when no disease was generated on board by hot, calm, and sultry weather; of course a much higher temperature in these respects is often experienced on board ships in the tropics; and the miasm and infectious air is often generated where no animal or vegetable putrefaction exists.

Infected vessels *do not always indicate their condition* by the disease developed *among their own crews or hands.* These may be perfectly seasoned to yellow fever miasm, by acclimation; or they may be natives of tropical latitudes, where yellow fever is indigenous, and consequently they may be protected against its influence by that kind of immunity.

Vessels have been known to leave West India ports, and after a long voyage, to arrive in ports of the United States, with all hands on board, in perfect health; and when none on board had suffered by yellow fever during the voyage. In these cases the infection had been carried into the hold of the ship in the form of *fomites*, by goods, beds, or other kind of freight, and, there confined, during the voyage in a close hot atmosphere, had become active and virulent. The hold being appropriated for freight alone, of course would be frequented little or none by the crew during the voyage, and would remain closed until the vessel arrived at her destined port. But so soon as she begins to discharge freight, the infected air is stirred up; and the hands and others passing into it are suddenly attacked with yellow fever and die. This has occurred too where no stench or putrid smell of any kind existed.

Dr. Bayley, for many years health officer of the port of New York, says: "I have known *many vessels* to arrive from ports where yellow fever prevailed, that were free from any unusually offensive smell, and their cargoes in a *sound state:* also vessels in *stone ballast*, from ports similarly circumstanced, *which were infected with the same contaminated air, that existed at the place they sailed from*, without its appearing to proceed from any foul materials generating it on board such vessels, that could be detected by the senses. The evidence of such infection was manifested not only by the crews and passengers, having died on board after leaving port; as these may have contracted the disease before they sailed; but such of the crews as had apparenly *resisted* the *malignant iufluence of such infected port*, sickened *and died of yellow fever, after they began to discharge the cargoes*, (which were in good condition) at the healthy port."*

In this case the infected cargoes confined in the holds generated a virulent infection throughout the hold of the ship during the voyage through the tropical seas. The crews and passengers not visiting the *hold*, of course escaped the disease, until the hatches were opened in the port of destination. Dr. Bayley states that "*in other cases vessels were infected;* but that the crews being seasoned, *or acclimated*, have arrived direct from an infected port, and *remained healthy* during the voyage, and *during the quarantine;* the vessels being apparently in proper condition, and having no sickness on board, nor any sensible bad air; but so soon *as other hands* came to assist, or to be on board, *they sickened and died of yellow fever*."†

Again Dr. Bayley declares that on the other hand he has witnessed at the New York quarantine ground, "that vessels have arrived from *healthy places* in tropical regions, with cargoes of *animal and vegetable matters in a state of putrescency*, with great foulness and the *extrication of much stench;* yet *no disease* existed among the crews or in those who assisted in discharging the cargoes."‡

Thus sustaining the doctrine, that the miasm or *infection of yellow fever* on *ship-board* as well as in cities and towns, is *entirely independent* of any *offensive smell*, or of any offensive or putrescent matters; and that those articles which throw off a putrescent stench are, in themselves, destitute of any power to produce yellow fever.

It is a point susceptible of proof, that towns and cities, even those in tropical latitudes, and upon the maritime borders of

---

*See Dr. Bayley's letter to Dr. Townsend, in Townsend on Yellow Fever, p. 92.

†See Townsend on Yellow Fever, p. 93.

‡Ibidem, p. 93–4.

the U. States, *are not liable* to or endangered by yellow fever epidemics, provided *they have no commercial intercourse* with West India, or *infected ports.* Those towns and ports, which have good deep harbors, and enjoy extensive trade with tropical ports, are liable to annual or occasional visitations of yellow fever as an epidemic. Those too which are remote, or inaccessible to shipping, are known to be habitually *exempt from yellow fever in any form.*

Notwithstanding the general predisposition, or "epidemic constitution of the atmosphere" occasionally exists from Boston and New York, along the whole seaboard of the Atlantic to St. Augustine, and along the whole Florida coast, as far as New Orleans, and even as far as the Colorado, in Texas; yet we see yellow fever prevailing in only a few of them, and in those only which enjoy a constant commercial intercourse by water with West India or infected ports. Hence it has prevailed in New York, Philadelphia, Baltimore, Norfolk, Charleston, Mobile, and New Orleans, while scores of other points intervening are exempt and healthy. Nor does it prevail simultaneously in all of these. Why has it not prevailed equally in Albany, Harrisburg, Fredericktown, Washington City, Richmond, Columbia, or Tuscaloosa? Simply because it cannot reach them.

All these places and hundreds more are exposed to the same "epidemic constitution of air," in hot, dry, sultry summers; yet only a few are exposed to the *indispensable requisite*, "*the exciting cause*"—the *leaven* of infected air from ships, and infected ports.

Dr. Rush and others of his school, have repeatedly admitted, that the impure or miasmatic air which accumulates in the holds of ships at sea in hot, sultry summers, *is morbific*, and that in an impure or contaminated air, it may act as "*an exciting cause*" to induce *yellow-fever* in those exposed to its influence. So far as the people of such town or port are concerned, it can surely make little difference, whether this "exciting cause" be imported from a West India port, or whether it be generated in the vessel only three days before her arrival. And, if it has the effect of producing a malignant epidemic which they might and would otherwise escape, it could certainly be very little consolation to them to learn, that it was not the exclusive cause, but only "*the exciting cause*" of the disease; or even that it was produced by putrescent matters as the predisposing cause. City authorities would do well to exclude *such exciting causes.*

The only point remaining to establish the *importability of yellow fever* is, to show that this infected air, miasm, or by whatever name it be called, is capable of diffusing itself into the surrounding atmosphere near wharves, and thus disseminating the disease. Besides the possibility of this mode of disseminating the disease among the crowds who daily frequent the immediate vicinity of the ship wharf, there are many others who certainly contract the disease by direct intercourse with the ship, as laborers, merchants' clerks, draymen, and the like.

There are many subtile gases in nature, especially in diseases, which possess the remarkable and mysterious property of penetrating to a great distance through the atmosphere, and of insinuating themselves and combining intimately with it, in a manner beyond our comprehension. The wonderful diffusion of aromatic particles, and of the minute particles of all volatile matters of smell are familiar examples. By what force or power does the putrescent particles of decomposing animal and vegetable matters mount the winds and force themselves into every man's olfactories, whether he will or not? Other effluvia, far less sensible to our organs, diffuse themselves no less extensively through the air, and are detected only by olfactory organs far more acute than ours. The delicate and to us imperceptible odor of a deer or other living animal is thrown into the air hundreds of yards from him, so as to direct the hound in the chase. Although this to the hound is sensible and plain, it entirely escapes our senses. Why then should we expect, with our blunt sense of smell, to trace the mysterious and insensible aura which is thrown off in disease?

The only point remaining to establish the importability of epidemic yellow fever in ports, is whether the *dissemination* of the infected air of the ship in the vicinity of the wharf, among the crowds that frequent them, and reside near them, together with ten, twenty, thirty, or more cases, which were contracted by going on board such vessels, be capable of causing the disease to spread, among those contiguous, who do not go on board, and this when the temperature of the season, and the sultry, and if desired the miasmatic state of the air, is in the most favorable condition for disseminating the disease. Of this we think there can be no reasonable doubt. Dr. Rush, and his coadjutors in favor of the domestic origin of yellow fever, admitted that it might, and had occasionally, spread from ships in an impure air.

Dr. Mitchell,* one of the most able advocates of the local or domestic origin of yellow fever, in a report made to Congress, February 25th, 1803, admits, and even contends that vessels often become highly infected while at sea; that an impure air is generated in the holds during *hot, sultry weather*, in tropical climates; that this infection may be communicated not only to those who go on board, but that the infected air may be *diffused in the atmosphere* about the wharves and shipping, and thus excite the disease more generally. He contends, very properly, against useless and idle detention at quarantine, when no disease or infection has been on board the vessel; but he urges the necessity of detention, and thorough ventilation and cleansing, in case of infection, until the infection is destroyed. In all this he denies the *foreign origin*, or infection. But is it not immaterial to those in sea port towns, whether the infection be generated on board, or contracted in a foreign port? If it can *spread* among those where the ship arrives, it is as important to guard against it as if it were of *foreign origin*. Dr. Rush unequivocally admits that there is much danger to be apprehended from "the foul air of ships," where cases of yellow fever have occurred. He also admonishes us, "to prevent the landing of *persons* af-

*See Med. Repos., vol. 6, p. 460, &c.

fected with the ship fever, in our cities, and the *more dangerous practice of ships pouring streams of pestilential air from their holds upon the citizens, who live near the docks and wharves.*" Med. Rep., vol. 6, p. 166.

Again, in a communication from Dr. Rush and others to Gov. Mifflin, in relation to the yellow fever of 1797, in Philadelphia and Kensington, it is maintained, "that, *in addition to* the filth and putrefaction about the city, *the foul air* issuing from the holds of two ships, (designated,) *produced the yellow fever*, independently of foreign contagion." Again, the authors declare, that "the close, unventilated holds of ships, after *long voyages in hot climates*, with perishable matters on board thrown open in a *heated*, *sultry atmosphere*, are a fruitful source of miasm: although they infer, that "yellow fever has not been *so often* propagated by *contagion*, as has been supposed." See Med. Repos. vol 2, p. 95, 96.

Without multiplying authority we will cite one case which is full of instruction to those who preside over the port police of our cities. We mean the "yellow fever" of New York in the summer and fall of 1822. This epidemic, if it could be so called, commenced by scattering cases from the 15th to the 20th of July, and cases multiplied gradually until the 15th of August, when it was considered epidemic. The whole number of yellow fever cases, from the 15th of July until the 1st of November when it ceased was about four hundred and thirty; of whom about two hundred and fifty died. It began in Rector street, near the wharf, where four ships' cargoes had been discharged from infected vessels a few days before. From this point it spread very slowly over several squares in the vicinity, having extended only a few squares in thirty days; while the remainder of the city was unusually healthy. The squares over which it prevailed most fatally, were bounded by wide, clean, and airy streets, and the most substantial buildings in the city; no filth could be found in the vicinity; the infected air from the original infected point having been wafted thither by the winds.

These are the facts without speculation, viz: the first cases began between the 15th and 20th of July. Between the 1st and the 9th of July, the cargoes of four infected vessels from Havana, were discharged at the wharf at the foot of Rector street, and stored in warehouses. Two of these vessels had lost some of their crews by yellow fever, on the voyage; the crews of the other two were Spaniards and acclimated sailors. During the first two weeks of July, the weather was very warm, the sun cloudless, and the air "*very calm and sultry.*" During the months of July and August, there were almost daily arrivals of other vessels from Havana, and other ports where yellow fever was known to be prevailing. During this period, the number of vessels from West India ports was unusual; because, on account of the terror of pirates, they came in companies, under the convoy of battle ships. The following are the arrivals at the quarantine ground, between the 11th of June, and the 17th of October, viz: From Havana, eleven vessels, having, or having had, on board forty-four cases of yellow fever and twenty one deaths; from Matanzas, one vessel, with three cases of yellow fever; from St. Jago, two vessels, having had three deaths from yellow fever; from Port au Prince, St, Domingo, four vessels, with six deaths from yellow fever; from Vera Cruz, between July 17th and 28th, three vessels, with nineteen cases and two deaths from yellow fever, before arrival. Besides these, there were, during that time, about forty other vessels from Southern and West India ports, whose crews, being Spaniards or acclimated seamen, had no cases during their voyage, although the air in some of the vessels proved infectious to those who were unacclimated.

If any one will take the pains to examine the accounts of this epidemic, as detailed by Dr. Townsend,* Dr. Bayley, and Dr. Walters, and after making every allowance, especially to Dr. Townsend for his views of contagion, of that charity which the advocates of the *exclusive local origin* of yellow fever are so ready to bestow, he will find ample reason to admit that the atmosphere about the wharves was *contaminated by the infectious air imported in ships from tropical climates.*

*See Med. Repos., vol. 4, p. 7 & 8; also, vol. 3, p. 46 &c., A. D. 1800.

A case presents directly in point, on this subject, as well as in relation to the agency of putrid exhalations and marsh miasm in the production of yellow fever. The town of Campeachy is situated on the Gulf of Mexico, in latitude 19° 45' N.—Vera Cruz is situated about three hundred and fifty miles distant, but in about the same latitude. Campeachy is healthy, and Vera Cruz is visited annually with yellow fever. The circumstances generally considered most favorable to the production of yellow fever, are equal in both places. Campeachy is built mostly of stone, upon a substratum of limestone rock; the soil of the surrounding country, as well as a part of the town, is a sandy loam, and often becomes very muddy; the town is surrounded by a stone wall about ten feet high; the streets are wide; the houses are large and airy; on the back part of the town, there is a high hill, or moderate mountain, which greatly interrupts ventilation, so that the inhabitants suffer greatly, from "all the inconveniences of a sultry, confined air." "In front of the middle of the city, is a large wharf or mole, extending one hundred yards into the water. Along this mole, there is constantly deposited large quantities of filth of every kind, together with "large quantities of *putrid fish*." "When the tide, (which rises and falls two or three feet,) retires, all these matters are left exposed in the mud, and on the shore, to the direct rays of a vertical sun, until the stench is intolerable to strangers." Besides these things, there are also other "accumulations of filth in other parts of the town." Yet "the inhabitants are very healthy;" there is only one physician in the place, and he has not half employment in his profession, although "the population is about ten thousand." (See Med. Rep. vol. 4, p. 5–8.) Vera Cruz is situated upon a sandy plain, with sand hills in its rear; contains no more filth than Campeachey; the air is no more confined or sultry, the inhabitants are no less temperate, yet Campeachey is healthy, and Vera Cauz is annually visited with yellow fever of the most malignant type. (See Med. Repos., vol. 4, p. 7 and 8; also, vol. 3, p. 46, &c. A. D. 1800.)

How is this paradox explained? Campeachey has an extensive, but shallow harbor, so that large ships cannot approach near the town; only boats and small craft can enter the harbor, such as do not exceed fifteen or twenty tons. Hence, so far as commerce with remote regions, and the foul air of large ships, from long tropical voyages, are concerned, it is equivalent to an inland town; and it is also free from the crowds of *strangers*, who infest large commercial ports. But Vera Cruz carries on an extensive commerce, has a fine deep harbor, and thus does not lack for infected air from ships, and crowds of strangers upon whom it operates These, when attacked, contribute to the infection, which is like "leaven" in the contaminated atmosphere, and assists in producing the epidemics. (Ibid, p. 78.)

Although the yellow fever is endemical all the year in Cuba, it is almost exclusively confined to the commercial ports. The elevated savannas of the interior are as much a stranger to it, as our own interior towns. In the ports, it attacks principally strangers, and prevails mostly from the beginning of July to the middle of November. When there is a great influx of strangers, it becomes more malignant, and finally attacks many of the acclimated inhabitants, especially such as are exposed to the ordinary exciting causes of fevers. It is believed, among the people of Cuba, that the influx of strangers, and their numerous attacks from yellow fever, contaminates the air, or infects it, and causes it sometimes to become epidemic, when otherwise it would not have prevailed.

(TO BE CONTINUED.)

